# Problems in Cancer

**Published:** 1902-07-05

**Authors:** 


### PROBLEMS IN CANCER.

The study of medicine in countries where, as in India, people of different race, of different customs and of different religions, live side by side exposed to the same climatic and such-like widespread influences, while differing greatly in regard to personal habits, offers constant opportunities for investigating the effect upon the human frame of certain customs peculiar to limited sections of the population. Take, for example, the incidence of cancer on different organs in different races. In the May number of the *Indian Medical Gazette*, Dr. Niblock has tabulated all the cases of cancer which have occurred in the Madras General Hospital during the last ten years, and he finds a curious thing. Of the 201 cases of epithelioma of the penis met with during that time, every one occurred in Hindus, and not one among Mohammedans. Yet putting on one side this particular form of carcinoma, cancerous disease was more common among the Mohammedans than among the Hindus. The reason of this freedom of the Mohammedan population from cancer of the penis is probably to be found in the practice of circumcision which obtains among them, and saves that organ from the chronic irritation which so often seems to play a part in determining the site at which the cancer process shall manifest itself. Exposure to traumatism may in the same way have to do with the fact that sarcomata affecting the lower extremity occur far more frequently in Hindus, who often go bare-footed, than in Mohammedans who more usually wear some form of protection to the foot. In reference to the oft-asserted relation between meat eating and the occurrence of malignant disease, the Indian statistics also have a word to say, for it appears that there is no great difference between the prevalence of such diseases as cancer and sarcoma in the meat-eating Mohammedans and the non-meat-eating Hindus.

